# A kinematic posture analysis of neurological assistants in their daily working practice-a pilot study

**DOI:** 10.1186/s12995-020-00286-9

**Published:** 2020-12-09

**Authors:** Anne Bijanzadeh, Ingo Hermanns, Rolf Ellegast, Laura Fraeulin, Fabian Holzgreve, Stefanie Mache, David A. Groneberg, Daniela Ohlendorf

**Affiliations:** 1 Institute for Occupational Medicine, Social Medicine and Environment Medicine, Goethe-University Frankfurt, Theodor-Stern-Kai 7, House 9b, 60590 Frankfurt am Main, Germany; 2 Institute for Occupational Health and Safety (IFA) of the German Social Accident Insurance (DGUV), Sankt Augustin, Germany; 3 Institute for Occupational Medicine and Maritime Medicine (ZfAM), University Medical Center Hamburg-Eppendorf (UKE), Seewartenstraße 10, House 1, 20459 Hamburg, Germany

**Keywords:** Neurology, Posture, Kinematic analysis, CUELA system

## Abstract

**Background:**

The aim of this pilot study was to analyze postures during the work of neurologists with respect to their occupational activities.

**Methods:**

A total data material of 64.8 h (3885.74 min) of nine (three m/six f) neurologists (assistant physicians) was collected. Kinematic data were collected using the CUELA system (electro-goniometry). In addition, the occupational tasks performed on-site were subject to a detailed objective activity analysis. All activities were assigned to the categories “Office activities” (I), “Measures on patients” (II) and “Other activities” (III). The angle values of each body region (evaluation parameters) were evaluated according to ergonomic ISO standards.

**Results:**

Only 3.4% of the working hours were spent with (II), while 50.8% of time was spent with (I) and 45.8% with (III). All tasks of category (II) revealed an increased ergonomic risk to the head, neck, trunk and back areas. During category (I) especially neck and back movements in the sagittal plane showed higher ergonomic risk levels.

**Conclusion:**

Despite frequently performed awkward body positions in (II), the ergonomic risk is considered as rather low, since the percentage time share totaled only 3.4%. As a result, “Office activities” have been detected as high predictor to cause stress load on the musculoskeletal system in the daily work of neurologists.

## Introduction

Not only in Germany, but also in other industrial countries [1, 2], studies on musculoskeletal disorders (MSD) have been conducted in various occupational sectors [3–10], for example, in the health sector [11–13]. In this sector, a high prevalence of musculoskeletal pain in the neck, shoulder and lower back region is observed [13, 14]. The main cause of MSD among healthcare professionals is the nature of their work; this requires excessive tension and concentration in activities which involve constantly lifting heavy objects, working for long periods of time in static standing positions or performing numerous repetitive tasks [15–20]. The latter have to be executed while writing medical records and patient documentations.

In recent years, the focus of research in this field has concentrated on ergonomic activities and processes within individual medical specialties such as surgery [21, 22] or gastroenterology [23]. In particular, during new surgical procedures [24] and new treatment techniques [25–28], musculoskeletal imbalances have been reported: 66–94% of surgeons report work-related MSD in open surgery [11, 21, 24, 29–31], 73–100% in conventional laparoscopy [32, 33], 54–87% in vaginal surgery and 23–80% in robotic surgery [25, 27, 28, 34–36]. In Italy, about 54% of 2041 sonologists [37, 38] stated that back, neck and shoulder pain, in particular, were the result of poor posture during work and led to an associated absence from work [39]; Similar results were also found in a study of 114 Canadian physicians [40]. Furthermore, 57% of physicians who perform regular ERCPs (endoscopic retrograde cholangiopancreatography) suffer from back pain, while 46% have complained with respect to neck pain [23, 41]*.* Regarding the influencing factors on MSD, some occupational factors (e.g., type of work, awkward postures, hard or accelerated work or stereotypical movements) may contribute [42–45]. Among these factors, working posture is the most important factor associated with the development of MSD [1, 46].

A substantial amount of time is spent on administrative tasks besides the direct patient care [47, 48]. Consequently, an increasing number of physicians are affected by physical inactivity at the workplace [49, 50] due to longer periods of computer work in the sitting posture. This occupational task is similar to usual office workers [51]. Mache et al. [52–57] have demonstrated that the activity profile of physicians has shifted to documenting on the computer, resulting in less time for direct patient contact. One group of physicians where this information is lacking, however, are the neurologists. Typically, neurologists must adopt awkward body positions due to the illnesses of their patients, e.g., stroke with hemiparesis or character changes with limited or absent willingness, or ability, to co-operate. During these activities, neurologists often have to lift, turn and support patients without any help from the nursing staff. Therefore, they often conduct unergonomic movement patterns, especially for diagnostic measures.

Therefore, the aim of this study was to collect sufficient kinematic data of the movement repertoire of neurologists during daily work in order to provide the first insight into the risk assessments of the work activities undertaken. The following hypotheses were tested:

- Hypothesis 1: The largest share of the total working time of neurologists is occupied by the category “Office activities” rather than “Measures on patients”.
- Hypothesis 2: Awkward posture patterns occur primarily in “Measures on patients”.
- Hypothesis 3: The highest ergonomic risk to the neck, torso and back areas occur during specific neurological activities (blood collection, lumbar puncture, placing intravenous catheters, examination).

## Material and methods

### MAGRO-MSA protocol

This study is part of the MAGRO-MSA study which has been established in 2015 [58]. In brief, a biomechanical measurement system for locomotor and posture analysis was integrated into a real-time medical job task analysis system termed MAGRO, which was described in greater detail earlier [59].

### Subjects

In order to obtain the target number of hourly material, nine (three male/six female) assistant physicians in neurology were measured. The recruitment was done by means of a presentation of the planned survey within a University Hospital and a municipal teaching hospital. Each of the nine physicians on the ward was accompanied for one working day during the entire shift. The weekend was omitted because of changes in the work structure (e.g., the physician supervises the ward alone or may have to work additionally in the emergency room). During data collection, the person collecting the data was placed at least two meters away and was not permitted to speak to the physician; this precaution was taken in order to minimize any influence on the physician’s behavior. The average age of the physicians was 32.1 ± 4.9 years and the statistical average of professional experience was 5.4 years ±5.6 years, while the weekly working time experienced was 51.6 h ± 8.7 h. The inclusion criteria included both female and male neurological residents. The exclusion criteria comprised activities in the functional department (department in which examinations such as EEG and sonography of the cervical and head vessels take place), intensive care unit and emergency room or if the physician had already experienced acute or chronic pain of the musculoskeletal system, however, since none of the neurologists had exceptional symptoms, this information was not integrated into the evaluation.

This study was approved by the Ethics Commission (135/14) of the Goethe University Frankfurt am Main. All participants signed, in advance, the required consent form for participation in the study.

### CUELA measuring system

The CUELA measurement system (IFA; Sankt Augustin/Germany) [60] (computer-assisted acquisition and long-term analyses of musculoskeletal loads) is a motion capturing system that records position and angle information via gyroscopes, acceleration sensors and potentiometers. In this way, a kinematic reconstruction of movement is possible. Since all sensors are connected via flexible cables, the respondent can easily perform all movement dimensions; this allows each individual body segment to be scanned in real time with a frequency of 50 Hz and an angular accuracy of 1° [12, 61].

For the measurements, the test person wears an upper body vest under their working clothes; the data storage unit of the posture system is attached to the back of the vest. The sensor system for measuring the lateral flexion and flexion of the upper body is located in the upper thoracic spine area, while the lumbar spine torsion is detected via a flexible shaft that merges into a lower sensor box. Additional sensors are attached to the extremities to measure flexion and extension movement. For the analysis of the cervical spine posture, the subject wears a headband with sensors that is connected to the upper sensor box of the thoracic spine.

### Mini-PC (objective activity analysis)

In order to obtain an analysis of the work processes of neurologists which is accurate to the second, special software was reprogrammed which can store manual inputs of real-time analyses for recording activity. Based on detailed analyses of the work spectrum, the computer program was modified so that the different categories of activity corresponded to the neurologist’s work. In this way, the individual activities can be named directly, and their duration recorded on the portable handheld computer at the same time.

To reflect all the activities of the everyday working life of the neurologist, three main categories were created: (1) “Office activities”, (2) “Measures on patients” and (3) “Other activities”. Within the 3 main categories; a total of 19 subcategories were distributed; this grouping serves to simplify similar movement patterns that can, thus, be compared. (Table 1).

**Table 1:** Presentation of all three main categories with the respective sub-categories and their explanations

Categories	Description of the Sub-categories
**Office activities**	
view file/documentation	documenting paper files at your desk
view file/PC	view file on PC
sort file	remove file from visit trolley and file papers
file entries	make arrangements
physician’s letter/final report	writing a doctor’s letter
morning meeting	sitting at the table and attending a meeting
PC work	diagnostic evaluation on the PC
radiographs	discuss radiological images with radiologists
telephone	telephone call via mobile phone, fixed network
**Measures on patients**	
blood sampling	draw blood from patient
conversation and notes/anamnesis	visit interview, admission interview with notes
hygiene	hand washing and disinfection
medical education	informing the patient about planned diagnostics
lumbar puncture	puncture of the spinal cord to obtain lumbar fluid
investigation	clinical examinations of the patient
placing of intravenous catheters	place the intravenous cannula to administer the infusion
**Other activities**	
conversation	conversations/discussions with relatives and colleagues
way	way to patients, doctor’s room, ward trolley
path and stairs	going for lunch, x-ray discussion, functional department

### Experimental procedure

For this field study, one working day of a stationary neurologist was randomly selected. The test persons wore the sensors of the CUELA system on the arms and legs, as well as on the thoracic and lumbar vertebrae under their clothing. In order to measure head movements, the study participant wore a headband with sewn-in sensors.

Parallel to the recording via the CUELA system, observers supported the participants and documented every movement of the neurologist on the Mini-PC. Using the software program developed especially for the CUELA system, the data could be synchronized with the CUELA system. All individual posture and movement patterns of the neurologists were thus visible in an angle-time diagram.

### Data evaluation

With the help of the CUELA software, the data of the activity analysis were synchronized with the CUELA measurement and, thus, a temporal allocation of the observed movement patterns and the associated activities was possible. Each work category was then separated into percentages according to relevance and duration. In order to compare the measured angles of the different activities, the percentiles 5 (P05), 25 (P25), 50 (P50 = median), 75 (P75) and 95 (P95) were used as output variables. This means that with a percentile of 5 (P05) of an activity, 5% of all measured angular values over time were below and 95% above the measured values. If one describes movements such as curvature forward, rotation or inclination to the right, this is represented by a positive sign and the opposite direction of movement by a negative sign. This is clearly visible in the lateral movement.

The angle values for each anatomical area (evaluation parameters) were evaluated according to the percentile intervals and according to the ergonomic aspects and were subsequently assigned to a color-coded angle area that represents the ergonomic standards (traffic light system: red/yellow/green) [62]. Based on the respective colors according to ISO standards, postures were classified as awkward, moderate or neutral [63–65]. Table 2 contains the body/joint angles according to the applied evaluation parameters and the evaluation criteria according to the ergonomic standards.

**Table 2:** Representation of the recorded body/joint angles according to the applied evaluation parameters and evaluation criteria, according to the ergonomic aspects

Body region	Joint / Body region	Direction of motion	Parameter (medical definition of degree of freedom)	Evaluation guideline values according to ISO 11226 and DIN EN 1005–4
head / neck	head	sagittal tilt	head tilt forward	neutral (green): 0° - 25° moderate (yellow): 25° - 85° awkward (red): < 0° & > 85°
		lateral tilt	head side tilt to the right	neutral (green): −10° - 10° awkward (red): < − 10° & > 10°
	cervical spine	flexion / extension	neck curvature to the front *[difference between head and thoracic spine.]*	neutral (green): 0° - 25° awkward (red): < 0° & > 25°
		lateral flexion	neck curvature to the right *[difference between head and thoracic spine.]*	neutral (green):-10° - 10° awkward (red): < −10° & > 10°
back	thoracic spine	flexion / extension	thoracic spine inclined to the front	neutra (green)l: 0° - 20° moderate (yellow): 20° - 60° awkward (red): < 0° & > 60°
		lateral flexion	thoracic spine inclined to the right	neutral (green): −10° to10° moderate (yellow): − 10° to −20° moderate (yellow): 10° to 20° awkward (red): < − 20° & > 20
	lumbar spine	flexion / extension	lumbar spine inclined to the front	no ISO standards available
		lateral flexion	lumbar spine lateral inclination to the right	
	torso	flexion / extension	back curvature to the front *[difference between thoracic and lumbar spine]*	neutral (green): 0° - 20° moderate (yellow): 20° - 40° awkward (red): < 0° & > 40°
			trunk inclined to the front *[average flexion of thoracic and lumbar spine]*	neutral (green): 0° - 20° moderate (yellow): 20° - 60° awkward (red): < 0°& > 60°
		lateral flexion	back curvature to the right *[difference between thoracic and lumbar spine]*	neutral (green): −10° - 10° moderate (yellow): − 10° - (−20°) moderate (yellow): 10° - 20° awkward (red): < − 20° & > 20°
			trunk inclined to the right *[average lateral flexion of thoracic and lumbar spine]*	
		torsion	back torsion to the right *[difference between thoracic and lumbar spine]*	

In addition to the angle values and the color-coded angles, the variance of movement of a certain activity was also evaluated. This was calculated using the modified interquartile range (mIR = [(P50-P05) + (P95-P50)]/2). The higher the value, the greater the variance of the movement.

For each sub-category, the “number” as well as the duration was first counted, e.g., how often and for how long telephone calls were made (175 times, 175.3 min). In order to gain a better overview, the number and duration of all sub-categories within a main category were combined to a total number and total duration; for both the total duration and the total number, the median and interquartile distances were calculated to determine the variance of the sub-categories within the main categories.

The Friedman test was then used to compare the number and duration of the three main categories and to check whether the individual areas of the main categories influence each other. Subsequently, the Wilcoxon post-hoc test was used to carry out the multiple group comparison, including the subsequent Bonferroni-Holm correction of the *p*-values. The significance was set at 5%. For this purpose, the data were averaged over all 9 neurologists. The mean values represent mean values over the individual activities of a category and not over the 9 participants of the study.

## Results

A total data material of 64.8 h (3885.74 min) was collected (excluding breaks and toilet visits because they were not related to the professional activity; often there were also no breaks). Therefore, nine neurologists were measured for an average of eight hours. Category I “Office activities” accounted for 50.8% (1972.99 min) of the working hours, with activities such as IT work, documentation of findings and therapy plans. “PC work” and “morning meeting” were responsible for the largest proportion of the time; both sub-activities together accounted for 47.5% (938.13 min) of total office time. In category II, “Measures on patients” accounted for 3.4% (131.03 min) of the working hours, e.g., with clinical examination and diagnostics such as lumbar puncture and intravenous catheterization. In category III, “Other activities” were responsible for 45.8% (1781.72 min) of the working hours, including activities such as conversations and visits to the patient or functional department; the task “conversation” accounted for the largest proportion of the time with 1363.55 min (76.5%).

### “Office activities” (I)

Table 3 contains all the descriptive angle data of category I.

**Table 3:**
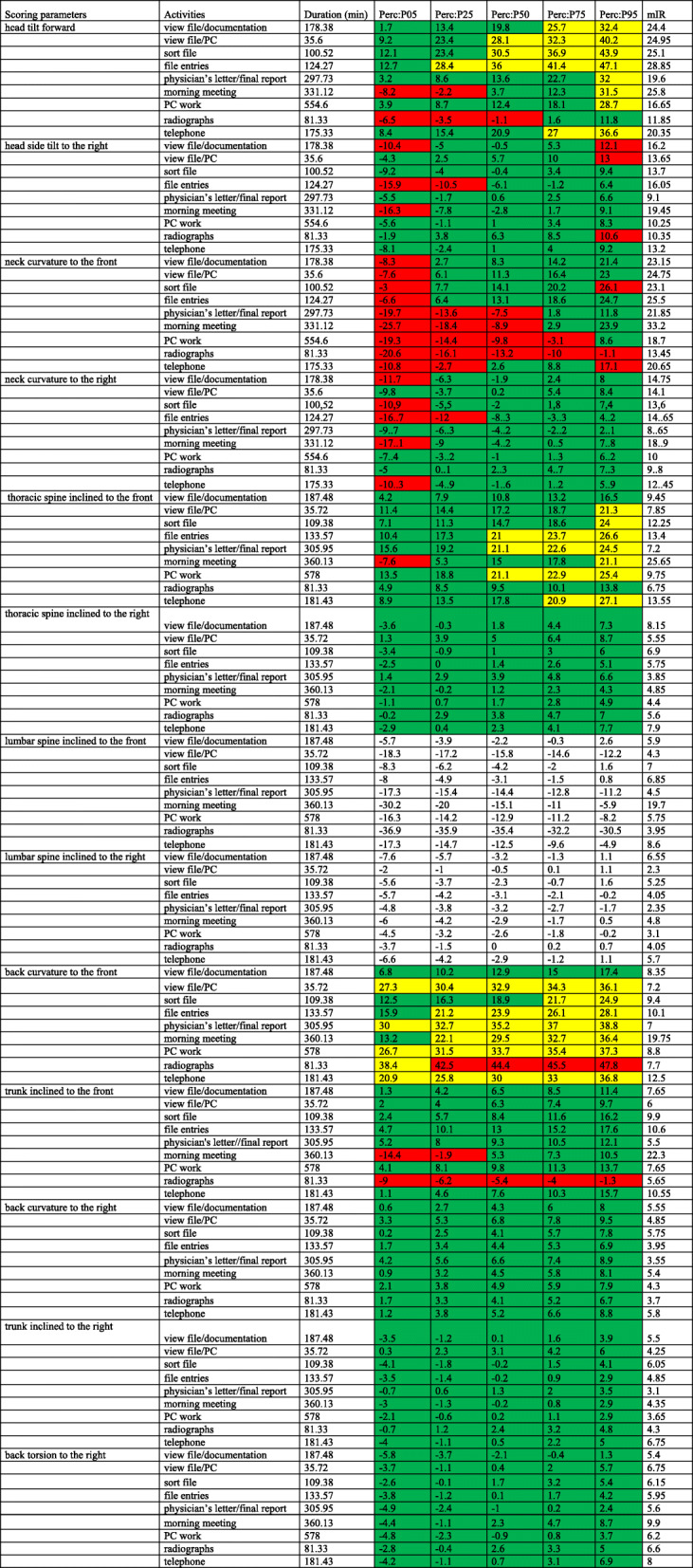
Office activities. Duration of the respective work steps, percentile values (P05, P25, P50, P75, P95) and values of the modified interquartile range (mIR)

In the head and neck region, the P50 data varied between − 1.1° and 36° (mIR 11.85° to 28.85°) for the head tilt forward, with the majority of activities being in the neutral range of angles. At P95 (11.8° to 47.1°) almost all sub-activities and at P75 (1.6° to 41.4°) the predominant portion of activities were in the moderate range of angles. The values of the “radiographs” were found to lay between P05 and P50 in the awkward range (− 6.5° to − 1.1°). For the head side tilt to the right, the angle values for P50 ranged from − 6.1° to 6.3° (mIR 9.1° to 19.45°) and for both P05 and P95 from − 16.3° to 13°. The P25, P50 and P75 values lay in the neutral range (− 7.8° to 10°), while the P05 and P95 values each had three values in the awkward range. In particular, the angle ranges at P05 of the sub-activities “view file/documentation”, “file entries” and “morning meeting” showed a stronger side tilt to the left (negative values indicate head sidetilt to the left while positive values indicate an side tilt to the right). On the other hand at “view file/documentation” did an awkward side tilt to the right exist at P95. With the neck curvature to the right, the angle values were between − 17.1° and 8.6° (mIR: 8.6° to 18.9°), with the exception of “file entries” (P25: − 11.6°), however, all sub-activities between P25 and P95 were found to occur in the neutral range. Except for the angle values of P05 (− 17.1° to − 5°), the predominant share of sub-activity (“view file/documentation”, “sort file”, “file entries”, “morning meeting” and “telephoning”) was carried out in the awkward range. In addition, these sub-activities were performed with a neck curvature to the left. For the neck curvature forward, the P50 values lay between − 10.2° and 14.5° (mIR: 13.45° to 33.2°). Both the P75 and P95 values were predominantly in the neutral range, with all sub-activities at P05 (− 25.7° to − 2.7°) and the majority of sub-activities at P25 (− 18.4° to 8.1°) being in the awkward range. The movement patterns of the neck curvature indicated a stronger neck reclination. The trunk inclined to the front showed that the P05 to P75 values were predominantly in the neutral range (− 7.6° to 23.7°; mIR 6.75° to 25.65°). In contrast, almost all P95 values (13.8° to 26.6°) were in the moderate range; in particular, the sub-activities “file entries”, “ physician’s letter/final report “ and “PC work” were found in the moderate range between P50 and P95. Only the “morning meeting” at P05 was in the awkward range (− 7.6°) and indicated a trunk reclination. Furthermore, the thoracic spine inclined to the right (− 3.6° to 8.7°; mIR 4.4° to 8.15°), back curvature to the right (0.2° to 11.9°; mIR 3.3° to 10.15°), back torsion to the right (− 5.8° to 8.7°; mIR 5.4° to 9.9°) and the trunk inclined to the right (− 4° to 6°; mIR 3.1° to 6.75°) all displayed angle values in the neutral range over all percentiles.

The symmetrical angle comparison of P05 (− 5.8° to − 2.6°) with P95 (1.3° to 8.7°) for the back torsion to the right revealed twice as high angle values for almost all sub-activities at P95 and, thus, indicated an increased back rotation to the right. A comparison of the angle values of P05 (− 3.6° to 1.4°) and P95 (4.3° to 8.7°) for the trunk inclined to the right conveyed a similar picture. In a symmetrical angle comparison, the trunk inclined was almost balanced, both to the right and to the left.

There exist no ergonomic standards for the lumbar spine (lumbar spine), however, all angle values between P05 and P95 for lumbar spine inclined indicated an increased retroflexion of the lumbar spine (− 36.9° to 2.6°; mIR 3.95° to 19.7°). The lumbar spine lateral inclination revealed a similar picture; here, an increased lateral inclination to the right (− 7.6° to 1.6°; mIR 2.3° to 6.55°) was found.

### “Measures on patients” (II)

Table 4 shows the sub-categories of “Measures on patients”.

**Table 4:**
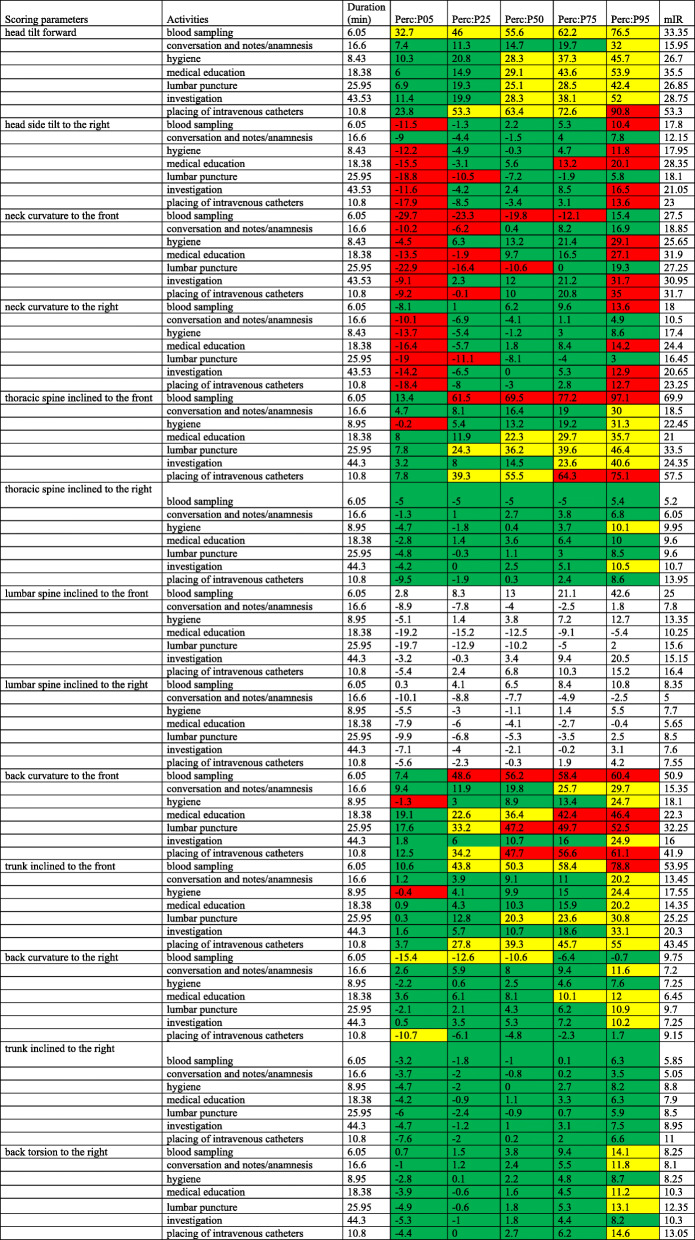
Measures on patients. Duration of the respective work steps, percentile values (P05, P25, P50, P75, P95) and values of the modified interquartile range (mIR)

In the head and neck area, the angle ranges between P50 and P95 for the head tilt forward were predominantly in the moderate range (14.7° to 90.8°; mIR 15.95° to 33.35°); in particular, “blood sampling” showed angles in the moderate range over all percentiles (32.7° to 76.5°; mIR 33.35°). The partial activity of “placing of intravenous catheters” was in the awkward range for P95, with an angle of 90.8°.

With the head side tilt to the right, the majority of the activities for P05 and P95 were in the awkward range.. The symmetrical comparison of the angle values between P05 and P95 were almost balanced between the sidetilt to the right and to the left. The exception to this was the “lumbar puncture” where the angle range of − 18.8° for P05 was three times as high as for P95 with 5.8°, thus, there was an increased head side tilt to the left.

Looking toward the evaluation parameters of the neck curvature, all angle values at P05 and the majority at P25 were in the awkward range (− 24.7° to 7.6°; mIR 18.85° to 31.9°).. At P95 (16.9° to 36°), “hygiene” (30.4°; mIR 25.65°), “medical education” (27.1°; mIR 31.9°), “examination” (32.3°; mIR 31°) and “placing of intravenous catheters” (36°; mIR 31.7°) were all found to be in the awkward range. A symmetrical comparison of the angle values between P05 and P95 showed that the P05 values for “blood collection” (− 24.7°) and “lumbar puncture” (− 22.9°) were higher than the P95 values (20.4° and 19.3°, respectively); this indicated an increased neck curvature backward (reclination). For all other sub-categories, it was the converse; here, there was an increased neck curvature forward.

With reference to the neck curvature to the right, almost all evaluation parameters at P05 were predominantly in the awkward range (− 19° to − 8.1°) and, occasionally, also at P95 (3° to 14.2°); these included “blood collection” (− 13.6° for P95), “medical education” (14.2° for P95), “examination” (13° for P95) and “placing of intravenous catheters” (12.7° for P95).

In a comparison of the angle values, the neck curvature to the right and to the left was balanced, except for the “lumbar puncture”. Here, the angle value at P05 (− 19°) was six times as high as at P95 (3°), which again indicated a stronger lateral inclination to the left.

The trunk inclined forward during “blood collection” had angle values in the awkward range (61.5° to 97.1°; mIR 69.9°) across all percentiles except P05, similarlythe sub-category “placing of intravenous catheters”. The values between P75 (64.3°) and P95 (75.1°) were in the awkward range. The remaining sub-activities medical educationwere found to have moderate angle ranges from P25 to P95 (24.3° to 46.4°; mIR 33.5°) and from P50 to P95 (22.3° to 35.7°; mIR 21°), respectively. A similar picture was obtained with the backcurvature to the front. Again, the activities “blood sampling” between P25 and P95 (48.6° to 60.4°; mIR 50.9°), “lumbar puncture” (47.2° to 52.5°; mIR 32.25°) and „placing of intravenous catheters “(47.7° to 61.1°; mIR 41.9°) between P50 and P95 were in the awkward range. The moderate range included “patient information“ (22.6°), “lumbar puncture“ (33.2°) and “placing of intravenous catheters “(34.2°) for P25 and “conversation/notes/anamnesis“ (29.7°), “hygiene“ (24.7°) and “examination” (25°) for P95.

Between P25 and P75 or P95, “blood collection” (43.8° to 58.4°, moderate up to P75; 78.8° awkward for P95; mIR 53.95°) and “placing of intravenous catheters “(27.8° to 55°; mIR 43.45°) were in the moderate range when the trunk inclined to the front .

The thoracic spine inclined to the right, back curvature to the right, back torsion to the right and the trunk inclined to the right showed body angles in the predominantly neutral range. The exception was P95 with predominantly moderate angular values.

Medical educationIn the symmetrical angle comparison, the “blood collection” (P05: -15.4°; P95–0.7°) was performed by an increased back curvature to the left. During the other activities, the back was more inclined to the right. A similar picture was obtained with the back torsion with predominant torsion to the right.

Regarding the lumbar spine inclined to the front (− 19.7° to 42.6°; mIR 7.8° to 16.4°) and the lumbar spine lateral inclination to the right (− 10.1° to 10.8°; mIR 5° to 8.35°), the high value differences between P05 for “blood collection” (2.8°), “hygiene” (− 5.1°), “examination” (− 3.2°) and “placing of intravenous catheters” (− 5.4°) and the P95 values (42.6°; 12.7°; 20.5°; 15.2°, respectively), indicated an increased lumbar spine inclined to the front. In comparison of the lumbar spine lateral inclination to the right, all activities except “blood collection” (P05 0.3°; P95 10.8°) showed a predominantly increased the lumbar spine lateral inclination to the left.

### “Other activities” (III)

Table 5 contains all angle values of category III.

**Table 5:**
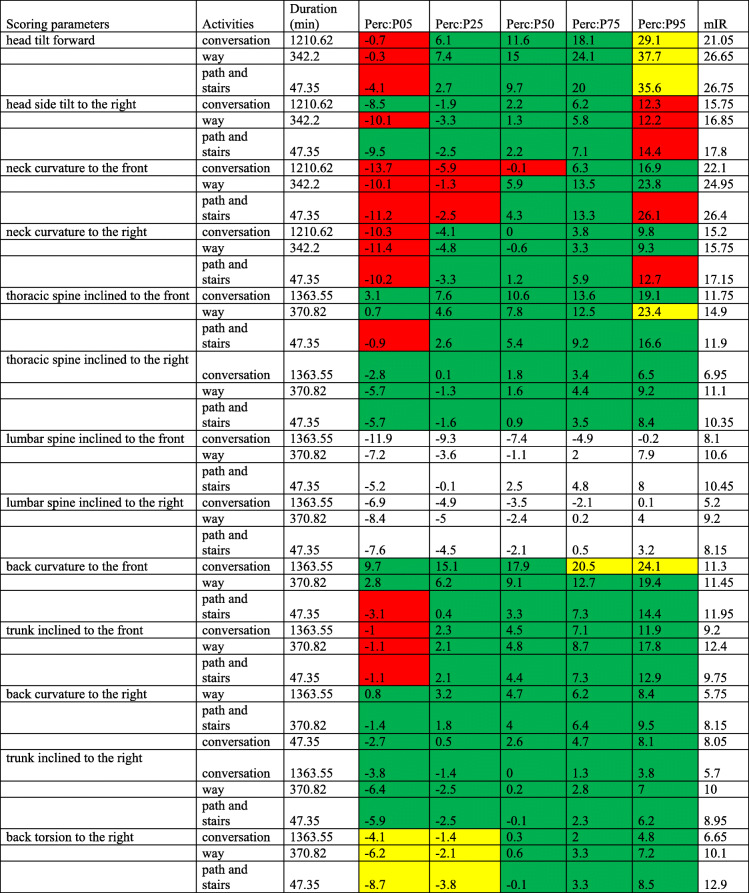
Other activities. Duration of the respective work steps, percentile values (P05, P25, P50, P75, P95) and values of the modified interquartile range (mIR)

All activities at P05 in the head and neck area were in the awkward range (− 4.1° to − 0.3°; mIR 21.05° to 26.75°) and at P95 in the moderate range (29.1° to 37.7°). In the case of the head side tilt to the right, “way” at P05 was − 10.1° in the awkward range. Furthermore, at P95 all activities laid in the awkward range (12.2° to 14.4°, mIR 15.75° to 17.8°). Turning attention to the neck curvature to the front, the awkward angular values ranged between P05 and P25 (− 1° to 13.5°; mIR 22.1° to 26.4°) across all sub-categories; only “path and stairs” exhibited some awkward angle values at P95 (26.1°). The symmetrical angle comparison showed half as high values for “way” and “path and stairs” between P05 (− 9.7° and − 11.2°, respectively) and P95 (24.1° and 26.1°, respectively). Awkward angle values could also be detected with neck curvature to the right at P05 (− 11.1° to − 10.2°; mIR 15.2° to 17.15°) and for trunk inclined to the front at P05 (− 1.1° to − 1°; mIR 9.2° to 12.4°). The neck curvature to the side was balanced in both direction. The situation was different for the trunk inclined to the front; the angle values for P95 were significantly higher (11.9° to 17.8°) than for P05 (− 1.1° to − 1°), if the P05 and P95 angles were compared. In addition, awkward angle values were detected for the trunk inclined to the front during “path and stairs” for P05 (− 0.9°) and also for the back curvature to the frontfor “path and stairs” at P05 (− 3.1°). Moderate angles were found in back torsion to the right between P05 (− 8.7° to − 4.1°; mIR 6.65° to 12.9°) and P25 (− 3.8° to − 1.4°); here, the symmetrical angle comparison between P05 and P95 was balanced. Furthermore, moderate angles were found between P75 (20.6°) and P95 (24.2°) when the back curvature to the front was during the sub-category “conversation”.

The thoracic spine inclined to the right, the back curvature to the right and the trunk inclined to the right showed neutral angle values between P05 and P95. With the exception of an increased back curvature to the right, with significantly increased values at P95 (8.7° to 9.8°) compared to P05 (− 1.9° to 0.6°), the other two body angles had a balanced ratio. During the lumbar spine lateral inclination to the right, high values were found at P05 (− 8.4° to − 6.9°) compared to P95 (0.1° to 4°); this resulted in a stronger lumbar spine lateral inclination to the left. With the lumbar spine inclined to the front, the angle values were approximately balanced except for the partial activity “conversation”. Here, the higher P05 value (− 11.9°), when compared to P95 (− 0.2°), indicated an increased lumbar spine extension.

### Comparison of the total number and total duration of all fields of activity

In order to quantify the differences between the differently investigated categories, interference statistical calculations now take place.

The total number (X^2^ (2) = 18.00, *p* <  0.001) and the total duration (X^2^ (2) = 14; p <  0.001) of the activities in the three fields of activity were statistically significant (Table 6). Furthermore, significant differences were found in the total number of activities in “Office activities” and in “Measures on patients” (*p* <  0.03), as well as between the total number of activities in “Office activities” and “Other activities” (*p* <  0.03). The mean total number of “Office activities” (median = 28.89, interquartile distance = 20.05 total number of individual activities) was, thus, significantly higher than the mean total number of “Measures on patients” (median = 3.5; interquartile distance = 3.33 total number of individual activities). The mean total number of “Office activities” was lower than the mean total number of “Other activities” (median = 71.5; interquartile interval = 40.25 total number of individual activities). The large interquartile distances were conspicuous in all activity categories. There were also statistically significant differences in the total duration of the activities. The Wilcoxon test confirmed characteristic differences in the total duration of “Office activities” and “Measures on patients” (*p* <  0.03). There were no significant differences found between the total duration of “Office activities” and “Other activities” (*p* <  0.86). The mean total duration for “Office activities” (median = 30.50, interquartile interval = 21.91 min) showed a significantly higher duration than the total duration for “Measures on patients” (median = 4.46, interquartile interval = 3.09 min). In comparison to the total number, the total duration of “Office activities” was higher than the total duration of “Other activities” (median = 25.46; interquartile distance = 13.84 min).

**Table 6:** Rank variance analysis (Friedman test and Wilcoxon post-hoc test)

Total number of activities	Chi^2^ *p*-value	Z	Wilcoxon-matched pairs *p*-value
Global test	0.001		
Office activities vs. Measures on patients		−2.67	0.01
Office activities vs. Other activities		−2.67	0.01
**Total duration of activities**			
Global test	0.001		
Office activities vs. Measures on patients		−2.67	0.01
Office activities vs. Other activities		−0.18	0.86

## Discussion

The collected data material of 64.8 h of the daily work routine of neurologists in Germany allows on the one hand an insight into specific work processes and means at the same time the first kinematic insight into neurological tasks on the other hand: 50.8% of all tasks were “Office activities”, while only 3.4% were “Measures on patients”. Hence, neurologists were exposed to the ergonomic risks associated with a patient examination to a very limited extent. The first hypothesis that the largest proportion of time is spent in the “Office activities” category can, therefore, be confirmed. Both the total number of activities nor the total duration in the “Office activities” were significantly higher than the “Measures on patients”. In addition, the large interquartile distances between the two main categories in total number and total duration indicated that there were inter-individual differences in the neurologists.

After the main category “Office activities” (I), “Other activities” (III) followed with 45.8%. The sub-category “conversations” should rather be counted as “Measures on patients”, since, for most of the time, the physician talked to the patient. However, “conversations” are not directly related to the “Measures on patients”, but rather function as accompanying activities for the “Measures on patients”.

In general, all “Measures on patients” had an increased ergonomic risk to the head, neck, trunk and back areas, since most activities were carried out standing next to the reclining patient, resulting in the neurologist’s head, neck and back being bent forward. In addition, the head and neck were tilted to the side during the conversation with the patient. Since the extent of the lateral inclination of the head and neck was approximately the same on both sides, it can be assumed that this depended on which side of the patient the neurologist was standing. This indicated an asymmetry during the activities. Specific neurological activities such as “blood sampling”, “placing of intravenous catheters” and “lumbar puncture” were the most common activities to lie in the moderate to awkward range with the highest awkward angle values. In particular, in the case of “blood collection”, the awkward angle values for the forward tilt of the head, trunk and back and extended neck were attributed to the awkward angle values required to interact with the patient lying down.

A further increased ergonomic risk to the head and neck area, especially in the lateral structures, occurred with the “lumbar puncture” activity, where the back and neck were held largely upright, but with an awkward lateral inclination. During the “lumbar puncture”, the pelvis was tilted backwards, and the trunk and back were bent forward up to awkward angle values. The reason for the increased ergonomic risk to the lateral head and neck area, as well as to the back, may be the fixed posture of the upper extremity during the puncture and/or the starting position of the patient, either in the sitting or lateral bent position. Furthermore, the inclination of the head and neck area to the left was compensated by a torsion of the back to the right; this would suggest an activity performed by right-handed persons. To what extent this movement is performed dynamically or statically and to what extent it affects the development of MSD must be analysed in further studies.

The comparison of the angle values between “Office activities” (category I) and category II (“Measures on patients”) showed that the activities in the office were predominantly carried out in the neutral range, whereas category II (“Measures on patients”) activities, on the other hand, were moderate to awkward. Here, however, what is conspicuous is the rather kyphotic sitting posture with predominantly moderate angles in the torso-back area with a forward inclination and an awkward forward neck curvature, in addition to the pelvis being predominantly tilted backwards. Ellegast et al. [66, 67] made similar observations on sitting postures at office and computer workstations; by using the same measuring system (CUELA), they documented for the P95 about 25° head inclination and 10° neck curvature forward. Also Mahammadipour et al. [51] confirms an increased prevalence of MSD in the lower back and neck of office workers. Neurologists measured an average of 33.8° for head inclination forward and 17.7° for neck curvature forward for the P95 during “office activities”. The comparison of both categories showed a clear difference between office and patient activity. Especially the differences in the head and neck area showed that the posture of the neurologist when acting on the patient was worse than when performing other activities. This is illustrated by the percentiles between P25 and P75. The average angles between the percentiles 25 and 75 for “Measures on patients”, with the head inclination forward and neck curvature forward, were rather in the moderate to strongly awkward range compared to “Office activities” which had rather lower angle values in the awkward range. The higher awkward angle values for “Measures on patients” were reflected with an average of 56.2° (P95 head tilt angle) or 25° (P95 neck flexion to the front) compared to 33.8° (P95 head tilt angle) and 17.7° (P95 neck flexion forward) at the edges of P05 and P95. Thus, hypotheses 2 and 3 can also be verified as awkward postural characteristics especially during patient activities, as well as during specific neurological activities, have been confirmed in this study.

However, the percentage of “Measures on patients” was significantly lower than that of “Office activities”, so it can be concluded that the ergonomic risk for the development of MSD during work on the patient is rather low. Consequently, the risk of developing MSD may be similar to that of office workers [51].

### Limitations

In addition, the Hawthorne effect [68] may have an effect on the results in that the participants can change their behavior as soon as they know that they are being observed. However, this effect should have had little influence on this study as the measurement was performed in a familiar working environment for at least 5 hours. Given this long measurement period, it is unlikely that participants would deviate from their usual working routine. In addition, the evaluator remained in the background during the measurements so that the participants were able to perform their tasks in a natural way. It should also be considered that wearing the vest with its sensors makes the test persons observe their posture. However, it cannot be assumed that the participants deviate from their usual working posture in view of the duration of the measurement.

Based on the collected data, further insights can be gained into the static posture and its effects during the daily work of a neurologist.

## Conclusion

Neurologists primarily perform “ Measures on the patient” in ergonomically awkward postures. However, since clinical investigations clearly account for the smallest proportion of typical activities performed during the working day, the ergonomic risk with regard to postures can be classified as low. In contrast, the increased proportion of “Office activities”, due to the high proportion of time spent in the sagittal plane, particularly neck and back movements, showed a similarly high ergonomic risk of developing MSDs as for office workers.

## Data Availability

The datasets used and/or analyzed during the current study are available from the corresponding author on reasonable request.

## Abbreviation

MSD: Musculoskeletal disorders
