# Correction: A kinematic posture analysis of neurological assistants in their daily working practice-a pilot study

**DOI:** 10.1186/s12995-023-00375-5

**Published:** 2023-06-20

**Authors:** Anne Bijanzadeh, Ingo Hermanns, Rolf Ellegast, Laura Fraeulin, Fabian Holzgreve, Stefanie Mache, David A. Groneberg, Daniela Ohlendorf

**Affiliations:** 1 Institute for Occupational Medicine, Social Medicine and Environment Medicine, Goethe-University Frankfurt, Theodor-Stern-Kai 7, House 9b, 60590 Frankfurt am Main, Germany; 2 Institute for Occupational Health and Safety (IFA) of the German Social Accident Insurance (DGUV), Sankt Augustin, Germany; 3 Institute for Occupational Medicine and Maritime Medicine (ZfAM), University Medical Center Hamburg-Eppendorf (UKE), Seewartenstraße 10, House 1, 20459 Hamburg, Germany

**Correction:** ***J Occup Med Toxicol 15, 36 (2020)***

10.1186/s12995-020-00286-9

In the original publication of Bijanzadeh et al. [1], the first names and surnames of five of the authors have been transposed. The correct names appear below:

Anne Bijanzadeh, Ingo Hermanns, Rolf Ellegast, Fabian Holzgreve, Daniela Ohlendorf

The author group has been updated above and the original article has been corrected.
